# Protease and Phytase Alleviate Digestive Limitations of Canola Seed in Broiler Chickens

**DOI:** 10.1002/vms3.71259

**Published:** 2026-10-01

**Authors:** Ramazan Sheikh‐Nazari, Kaveh Jafari‐Khorshidi, Mohammad Ali Jafari

**Affiliations:** ^1^ Department of Animal Science QaS.C., Islamic Azad University Qaemshahr Iran

**Keywords:** digestibility, digestive enzyme, microflora, morphology, viscosity

## Abstract

**Background:**

While canola seed is a valuable protein and energy source in poultry nutrition, its use is often limited by antinutritional factors that impair nutrient utilisation and bird performance.

**Objectives:**

This study investigated the effects of phytase and protease enzymes in diets containing canola seed on performance, nutrient digestibility, digestive enzyme activity, intestinal morphology, ileal microbial populations, intestinal viscosity and tibia bone mineralisation in broiler chickens.

**Methods:**

A total of 300 male Ross 308 one‐day‐old chicks were used in a completely randomised design with 5 treatments, 5 replicates and 12 chicks per replicate. The experimental treatments included: control: a corn–soybean meal‐based control diet without canola seed; CS‐10: a diet containing 10% canola seed; CS‐PRO: a diet containing 10% canola seed supplemented with protease; CS‐PHY: a diet containing 10% canola seed supplemented with phytase and CS‐PRO‐PHY: a diet containing 10% canola seed supplemented with a combination of protease and phytase.

**Results:**

Feed intake and body weight gain were significantly higher in the control and CS‐PRO‐PHY treatments at different experimental periods compared to the CS‐10 diet (*P* < 0.05). Apparent metabolisable energy and the digestibility of crude fibre and ash were higher in the control group and in broilers fed CS‐PHY and CS‐PRO‐PHY compared with birds fed the CS‐10 and CS‐PRO diets (*P* < 0.05). At Day 10, amylase and lipase activity were highest in broilers fed the CS‐PRO‐PHY (*P* = 0.001). The villus height and villus height‐to‐crypt depth ratio on Days 10 and 42 were significantly higher in broilers fed the control diet, the CS‐PRO‐PHY and CS‐PHY compared to the CS‐10 (*P* < 0.01). At Day 10, *Lactobacillus* populations were highest in broilers fed the CS‐PRO‐PHY and the control diet, and lowest in the CS‐10 (*P* = 0.03). Duodenal and jejunal digesta viscosity was significantly higher in broilers fed the CS‐10 diet compared with all other treatments (*P* < 0.01). Tibia weight, length and weight‐to‐length index of the bone in the CS‐PRO‐PHY treatment were significantly greater than those in the CS‐10 and CS‐PRO treatments (*P* < 0.05). Moreover, bone strength in the control, CS‐PRO‐PHY and CS‐PHY treatments was higher than in the CS‐10 treatment (*P* = 0.034).

**Conclusions:**

Supplementation of broiler diets containing 10% canola seed with protease and phytase, particularly in combination, effectively mitigated the negative effects of canola on nutrient digestibility, intestinal morphology, microbial balance, digesta viscosity and bone quality, leading to restoration of performance.

## Graphical abstract

Protease and phytase, especially in combination, counteracted the digestive limitations of 10% canola seed in broiler diets by improving nutrient utilisation, intestinal health, microbial balance, digesta viscosity, bone quality and growth performance.

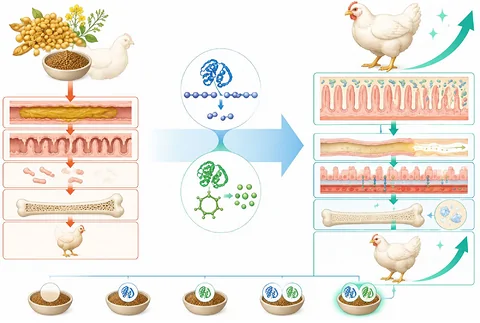

## 1 Introduction

Soybean meal remains the predominant protein source in broiler nutrition due to its high crude protein (CP) content and favourable amino acid profile, and is typically included at 20% to 30% of the diet. As a major co‐product of soybean oil extraction, the availability and price of soybean meal are influenced by global market dynamics (Pope et al. 2023). Nevertheless, price volatility, supply chain instability and sustainability concerns have intensified interest in identifying alternative protein ingredients, particularly in regions that depend on imported soybean meal (Da Silva et al. 2010). Among potential alternatives, canola products, including canola seed and canola meal, have attracted considerable attention (Kandel et al. 2025a); however, their inclusion in broiler diets remains limited because of their potential to impair growth performance, particularly during the early stages of life (Ahmed et al. 2014; Rad‐Spice et al. 2018). Inclusion levels are generally restricted to a maximum of 80 g/kg in starter diets and 120 to 130 g/kg in finisher diets. The use of canola seed in poultry diets is constrained by its nutrient composition and the presence of antinutritional factors, including tannins, lignin, phytate and glucosinolates, which may negatively affect feed intake (FI) (Khajali and Slominski 2012; Barekatain et al. 2015; Toghyani et al. 2017).

Supplementation with exogenous enzymes, including phytase and protease, has been shown to mitigate some of these limitations (Wang et al. 2026). Newkirk and Classen (2001) reported that both crude and purified phytase improved the apparent metabolisable energy (AME) in corn–canola meal‐based diets. Proteases have also been shown to enhance protein hydrolysis and amino acid absorption in broiler chickens (Liu et al. 2013), indicating their potential value in diets containing canola seed. Supporting this notion, Cowieson et al. (2019) reported significant increases in AME and net energy following the addition of protease to broiler diets containing 7.9% canola meal. More recently, Wang et al. (2026) reported that protease supplementation produced greater benefits in corn–canola meal diets than in corn–soybean meal diets, improving growth performance and nutrient digestibility. In another study, replacing soybean meal with canola seed and canola meal at increasing inclusion levels reduced FI by 6.90% and BWG by 7.11% at the highest inclusion level compared with a diet without canola products (Kandel et al. 2025b). The authors suggested that the reduction in growth performance was primarily attributable to decreased FI, as canola inclusion did not significantly affect feed conversion ratio (FCR) or nutrient digestibility coefficients (Kandel et al. 2025b). Kasayizadeh et al. (2025) also reported increased *Lactobacillus* populations, reduced *Escherichia coli* counts in the ileum and improved intestinal morphology in broiler chickens fed canola seed. However, studies evaluating the interaction between canola seed and the exogenous enzymes phytase and protease remain limited.

Accordingly, the present study investigated the individual and combined effects of phytase and protease on growth performance, nutrient digestibility, intestinal microbial populations, intestinal morphology and intestinal viscosity in broiler chickens fed diets containing canola seed. Particular emphasis was placed on evaluating phytase‐only and protease‐only treatments to address practical considerations faced by commercial nutritionists. Given the volatility in the prices of protein‐rich feed ingredients, industry stakeholders are especially interested in determining which enzyme (phytase, protease or their combination) provides greater improvements in growth performance and nutrient digestibility when only one option is economically justifiable. Therefore, both protease and phytase were included in this study to assess their value relative to combined enzyme supplementation in diets containing canola seed.

## 2 Materials and Methods

### 2.1 Birds, Treatments and Management

A total of 300 one‐day‐old male Ross 308 chicks (mean body weight of 44.8 ± 0.31 g) were used in a completely randomised design with 5 treatments, 5 replicates and 12 chicks per replicate. The experimental treatments included: control: a corn–soybean meal‐based control diet without canola seed; CS‐10: a diet containing 10% canola seed; CS‐PRO: a diet containing 10% canola seed supplemented with protease; CS‐PHY: a diet containing 10% canola seed supplemented with phytase and CS‐PRO‐PHY: a diet containing 10% canola seed supplemented with a combination of protease and phytase. Protease (Idozyme X‐3002 protease 20000 U/g, Sabz Andishan Bartar Co., Tehran, Iran) and phytase (Microtech phytase 10000 FTU/kg, Sabz Andishan Bartar Co., Tehran, Iran) were included at 0.15 and 0.05 g/kg, respectively, in experimental diets. The birds were housed in floor cages measuring 100 cm length × 70 cm width × 60 cm height. The lighting program was maintained at 23L:1D for the first 7 days, followed by 18L:6D for the rest of the experimental period. Relative humidity was maintained at approximately 50% throughout the experimental period. Ambient temperature was adjusted according to broiler age based on the Ross 308 management recommendations (Ross 308 Broiler Management Handbook 2022 EN—Aviagen). All experimental diets were provided during the starter (1–10 days), grower (11–24 days) and finisher (25–42 days) periods (Table 1). The birds had free access to feed and water. Vaccination against Newcastle disease was conducted via drinking water on days 7, 17 and 28, and against infectious bursal disease on Day 14. The chemical composition of canola seed (Dry matter [DM**]**, CP, ash and ether extract [EE]) was analysed according to the AOAC (2006) procedures, and crude fibre (CF) was determined by the methods of Crampton and Maynard (1938). The glucosinolates in canola seed materials were determined by gas liquid chromatography of trimethylsilyl derivatives of desulphated glucosinolates according to the Raney and McGregor method (1983). The method was standardised using BC 190 reference material, and the results were comparable to those obtained by high‐performance liquid chromatography (HPLC). All analyses were performed in duplicate (Table 2).

**TABLE 1:** Components and chemical compositions of the diets during starter, grower and finisher periods.

Diet ingredients (%)	Starter (Days 1–10)		Grower (Days 11–24)		Finisher (Days 25–42)	
	Control	Canola	Control	Canola	Control	Canola
Corn	58.32	56.43	60.85	57.11	63.50	63.51
Soybean meal (44%)	34.03	27.56	31.04	27.07	28.12	19.32
Canola seed (21.25%)	−	10.00	−	10.00	−	10.00
Soybean oil	1.60	−	2.35	−	3.00	1.60
Dicalcium phosphate	1.90	1.90	1.90	1.90	1.80	1.90
Calcium carbonate	1.40	1.40	1.40	1.40	1.40	1.40
Salt	0.25	0.25	0.21	0.25	0.10	0.10
Bentonite	1.00	1.00	1.00	1.00	1.00	1.00
Vitamin premix^a^	0.50	0.50	0.40	0.40	0.30	0.30
Mineral premix^b^	0.50	0.50	0.40	0.40	0.30	0.30
DL‐Met	0.20	0.17	0.15	0.17	0.17	0.20
L‐Lys HCl	0.25	0.24	0.25	0.25	0.26	0.27
L‐Thr	0.05	0.05	0.05	0.05	0.05	0.10
Total	100.00	100.00	100.00	100.00	100.00	100.00
**Calculated composition**						
ME (kcal/kg)	2860	2860	2920	2920	3000	3000
Crude protein (%)	20.40	20.60	19.00	19.00	17.00	17.00
Ether extract (%)	2.35	4.27	2.52	4.27	2.48	4.29
Crude fibre (%)	3.70	3.20	3.30	3.00	3.50	2.80
Linoleic acid (%)	1.37	2.19	1.37	2.20	1.34	2.20
Calcium (%)	1.06	1.04	1.06	1.04	1.06	1.04
Available phosphorus (%)	0.46	0.44	0.45	0.44	0.43	0.42
Sodium (%)	0.18	0.16	0.16	0.17	0.16	0.16
Digestible methionine (%)	0.56	0.52	0.48	0.48	0.46	0.45
Digestible methionine + cysteine (%)	0.89	0.86	0.80	0.80	0.75	0.75
Digestible lysine (%)	1.26	1.26	1.18	1.23	1.21	1.11
Digestible threonine (%)	0.86	0.87	0.81	0.80	0.72	0.70
Digestible arginine (%)	1.35	1.38	1.26	1.34	1.18	1.17

^a^ Provides the following per kg of diet: 4.13 mg retinol, 60.00 µg chole‐calciferol, 30.00 mg Dl‐α‐tocopherol, 3 mg menadione, 2.20 mg thiamine, 8.00 mg riboflavin, 5.00 mg pyridoxine, 11.00 µg cyanocobalamin, 1.50 mg folic acid, 150.00 µg biotin, 25.00 mg calcium pantotenate and 65.00 mg nicotinic acid.

^b^Provides the following per kg of diet: 60.00 mg Mn (manganese sulphate), 40.00 mg Zn (zinc oxide), 0.33 mg I (potassium iodate), 80.00 mg Fe (ferrous sulphate), 8.00 mg Cu (copper sulphate), 0.15 mg Se (sodium selenite) and 150.00 mg ethoxyquin.

**TABLE 2:** Analysed chemical composition of canola seed samples (*n* = 10).

Composition %	Canola seed
Dry matter	93.37
Crude protein	21.25
Ether extract	42.76
Crude fibre	18.39
Ash	4.35
Calcium	0.38
Phosphorus	0.57
Phytate phosphorus (mg/kg)	5264
Tannins	0.36
Glucosinolate (μ mole/g)	9.37
Gross energy (kcal/kg)	7063

All the values are analysed at the viromed Central Analytical Laboratory, Pardis Technology Park, Tehran, Iran.

### 2.2 Performance

To calculate the amount of FI of each repetition, the amount of feed remaining at the end of each period was deducted from the total feed given during the period. On Days 1, 10, 24 and 42, all chickens of each experimental unit were weighed as a group. To calculate the BWG of each repetition in each period, the difference between the final weight and the beginning of the breeding period was determined. The FCR was also calculated by dividing the average FI by the average BWG of chickens for each period (Hassanlou et al. 2026).

### 2.3 Digestibility Assay

To determine apparent ileal digestibility coefficients of nutrients, on Day 3 before ileal digesta collection (Day 39), 5 g of titanium dioxide per kg feed was added to the diet as an exogenous marker (Ebrahimi et al. 2025). At the end of the experiment (on Day 42), two birds per replicate were euthanised by CO_2_ asphyxiation, and the contents from the distal portion of the ileum, spanning from Meckel's diverticulum to the ileo‐cecal‐colonic junction, were obtained by flushing with distilled water into plastic containers. The diets and ileal contents were oven‐dried (60°C for 72 h) and then left in the open air for 24 h to equilibrate. The diets and ileal contents were ground (<0.75 mm) and the DM, CP, EE and CF contents were measured according to standard methods (AOAC 2006). The gross energy of the experimental diets and ileal contents was determined using a bomb calorimeter (Parr 6200 bomb calorimeter, Parr Instruments Co., Moline, IL) with benzoic acid as the calibration standard. The optical absorbance of the samples was measured by a spectrophotometer (Spectronic 21D; Milton Roy Co., Rochester, NY) at a wavelength of 440 nm, and the amount of titanium oxide in the samples was determined by comparing it with a standard curve (Short et al. 1996).

Apparent total‐tract nutrient digestibilities were calculated by the index method, which follows the equation described by Kong and Adeola (2014):

Apparent nutrient digestibility (AND, %)  =  [1 – (*T_i_* ÷ *T_o_*) × (*X_o_* ÷ *X_i_*)] × 100

 where AND is the apparent total‐tract digestibility of DM, CP and EE expressed as a percentage; *T_i_* represents the concentration of titanium (g/kg DM) in experimental diets and *T_o_* represents the concentration of titanium (g/kg DM) in excreta output; *X_i_* and *X_o_* are the concentrations of nutrients in experimental diets and excreta output, respectively. The AME was calculated using the following equations (Rahbari et al. 2025):

AME (kcal/kg) = GE diet – (GE feces × Titanium dioxide diet / Titanium dioxide feces)

### 2.4 Digestive Enzyme Activity

On Days 10 and 42, jejunal digesta samples were obtained from two birds per replicate. A subsample (0.1 g) was homogenised with 900 µL of sterile saline solution and kept at 4°C. The homogenates were thoroughly mixed using a vortex and subsequently centrifuged at 3000 rpm for 10 min to obtain the supernatant. The activities of amylase, lipase and total protease in the supernatant were quantified using chicken‐specific ELISA kits (amylase: DDP01155‐N; lipase: DDP01184‐S; total protease: DDP01271‐M; Delta Darman Part Co., Tehran, Iran) according to the manufacturer's instructions (Guo et al. 2025).

### 2.5 Intestinal Morphology

At 10 and 42 days of age, the jejunum of two birds from each replicate (10 for each treatment) was dissected for morphological evaluation and analysed according to the method of Liu et al. (2022). Briefly, 1 cm of the jejunum was removed and washed with 0.9% saline to remove the contents. All samples were fixed in 10% buffered formalin for histological evaluation. Intestinal morphological measurements included villus height, villus width and crypt depth in each section. The villus height‐to‐crypt depth (VH/CD) ratio was then estimated by dividing villus height by crypt depth (Hassanlou et al. 2026).

### 2.6 Ileal Microbiota

At 10 and 42 days of age, the gastrointestinal tracts of 10 birds per treatment were dissected, and ileal contents were aseptically collected into sterile containers and stored at −20°C until analysis. Ileal digesta were sampled from the region extending from Meckel's diverticulum to the ileo‐cecal junction. Approximately 1 g of ileal content was suspended in 9 mL of sterile 0.9% saline solution and homogenised, followed by preparation of appropriate serial dilutions. *Lactobacillus* spp.*, Clostridium perfringens* and *E. coli* populations were enumerated using de Man, Rogosa and Sharpe (MRS) agar, sulphite iron agar (SIA) and MacConkey agar (Merck, Darmstadt, Germany), respectively. Microbial counts were expressed as log10 colony‐forming units (CFU) per gram of ileal digesta (Althagafi et al. 2025).

### 2.7 Intestinal Viscosity

To assess intestinal digesta viscosity, two broiler chickens from each replicate were euthanised, and digesta samples were collected (10 and 42 days). The small intestine was segmented into the duodenum (duodenal loop), jejunum (from the duodenal loop to Meckel's diverticulum) and ileum (from Meckel's diverticulum to the ileo‐cecal junction). Digesta from the duodenum, jejunum, ileum and cecum were harvested for viscosity determination. Approximately 1.5 g of digesta from each intestinal segment was divided into two subsamples, transferred into microtubes and centrifuged at 12,700 rpm for 5 min. Following centrifugation, about 0.5 mL of the supernatant was collected, and digesta viscosity was measured using a Brookfield digital viscometer (model I‐LVDV) as described by Gouran et al. (2025).

### 2.8 Tibial Bone Characteristics

On the final day of the experiment, both the left and right tibiotarsi were harvested from each bird. To facilitate the removal of soft tissue, the bones were labelled, placed in plastic bags and immersed in boiling water (100°C) for 10 min. After cooling to room temperature, the bones were manually defleshed, the patella was removed and the samples were air‐dried for 24 h. The weight, length and diaphysis diameter (at the midpoint) of the tibiotarsi were recorded using a high‐precision digital balance (0.01 g; Model K‐E‐B‐602, Kerona, Shanghai, China) and a digital caliper (0.01 mm; Bakingwin, Shenzhen, China). The strength of the tibia of the left leg against pressure was measured in newtons (Ebrahimi et al. 2025). The strength test was performed using a Santam device (Model DBBP‐500, Santam Engineering Co., Tehran, Iran) at 60.00 mm per min. The resulting bone fragments were subsequently used for chemical analysis. For ash determination, bone fragments were dried in a forced‐air oven at 150°C for 24 h and then incinerated in a muffle furnace at 600°C for 6 h. The ash content was calculated as a percentage of the dry bone weight (AOAC 2006). Calcium concentration in tibia samples was quantified by flame atomic absorption spectrophotometry (Varian Spectr AA 50B Atomic Absorption Spectrometer; Varian Ltd., USA) in accordance with AOAC (2006) procedures. Tibial phosphorus content was also determined spectrophotometrically using a Jenway Genova MK3 spectrophotometer (Jenway, UK) following the analytical methods outlined by AOAC (2006). The contralateral tibiotarsus was cross‐sectioned at the midpoint of the diaphysis. The thickness of the medial and lateral walls was measured using digital calipers and DinoCapture 2.0 software. The medullary canal diameter was calculated by subtracting the sum of the medial and lateral wall thicknesses from the total diaphysis diameter. To evaluate bone health and mineralisation, the following indices were calculated (Çapar Akyüz et al. 2024): Weight‐to‐length index = Bone weight/Bone length (an indicator of bone density).

$$\begin{array}{ccc} & & \mathrm{Tibiotarsal}\;\mathrm{index}\;\;\left(\%\right)\\ & & =\frac{\mathrm{Diaphysis}\;\;\mathrm{diameter}-\mathrm{Medullary}\;\;\mathrm{canal}\;\;\mathrm{diameter}}{\mathrm{Diaphysis}\;\;\mathrm{diameter}}\;\times 100 \end{array}$$

$$\mathrm{Bone}\;\;\mathrm{strength}\;\;\mathrm{index}=\frac{\mathrm{Trbial}\;\mathrm{length}}{\sqrt[{3}]{}\mathrm{Tibial}\;\mathrm{weight}}$$

### 2.9 Statistical Analysis

The obtained data were tested for normality using the PROC UNIVARIATE of SAS, version 9.2, and whenever needed, percentage data were transformed using the ArcSin√x transformation. The data were analysed using the PROC GLM of SAS (version 9.2). Differences among means were determined using Duncan's test, and *P* values <0.05 were considered statistically significant. The statistical model of the current study was as follows:

Y_ij_ = μ +T_i_ + ε_ij_where *Y_ij_*, *μ*, *T_i_* and *ε_ij_* represent the observation, the mean of observations, the treatment effect and the experimental error of each observation, respectively.

## 3 Results

### 3.1 Broiler Performance

The effect of protease and phytase enzymes in diets containing canola seed on the performance of broilers is reported in Table 3. FI was significantly higher in the control and CS‐PRO‐PHY treatments at different experimental periods compared to the CS‐10 treatment. However, in the period of 1 to 10 days of age, the CS‐PRO and CS‐PHY treatments, although showing lower FI than the control treatment and CS‐PRO‐PHY, had higher FI compared to the CS‐10 treatment. BWG during the starter (1–10 days), grower (11–24 days) and overall experimental (1–42 days) period was greater in the control group and in birds fed the CS‐PRO‐PHY diet than in those fed the CS‐10 diet (*P* < 0.05). During the grower phase, broilers receiving the CS‐PHY diet exhibited lower weight gain than the control and the CS‐PRO‐PHY treatment, while still achieving higher BWG compared with the CS‐10 diet (*P* = 0.003). During Days 11 to 24, a lower FCR was observed in the control group and in broilers fed the CS‐PRO‐PHY compared with birds fed CS‐10, CS‐PRO and CS‐PHY (*P* = 0.037).

**TABLE 3:** The effect of protease and phytase enzymes in diets containing canola seed on the performance of broiler chickens.

Items	CON	CS‐10	CS‐PRO	CS‐PHY	CS‐PRO‐PHY	SEM	*P*‐value
**Average feed intake, g/day**							
1–10 days	34.93^a^	28.93^c^	32.64^b^	33.58^b^	34.21^a^	0.79	0.001
11–24 days	86.76^a^	79.90^c^	82.19^b^	83.36^a,b^	85.33^a^	1.18	0.008
25–42 days	164.72^a^	150.61^b^	157.78^a,b^	161.35^a,b^	165.35^a^	2.16	0.036
1–42 days	107.83^a^	98.07^b^	102.79^a,b^	104.93^a,b^	107.45^a^	1.43	0.001
**Body weight gain, g/day**							
1–10 days	28.50^a^	25.36^b^	26.06^a,b^	26.56^a,b^	27.19^a^	0.48	0.014
11–24 days	63.98^a^	53.12^c^	54.85^b,c^	58.84^b^	62.91^a^	1.06	0.003
25–42 days	82.7	79.92	80.76	81.81	82.42	1.84	0.155
1–42 days	63.56^a^	58.00^b^	59.10^b^	61.00^a,b^	62.77^a^	0.87	0.048
**Feed conversion ratio**							
1–10 days	1.23	1.2	1.25	1.26	1.26	0.03	0.282
11–24 days	1.36^b^	1.50^a^	1.50^a^	1.42^a,b^	1.36^b^	0.06	0.037
25–42 days	1.99	1.91	1.95	1.97	2.01	0.08	0.196
1–42 days	1.7	1.69	1.74	1.72	1.71	0.04	0.314

The experimental treatments included: CON = control diet; CS‐10 = diet containing 10% canola seed; CS‐PRO = canola seed diet supplemented with protease; CS‐PHY = canola seed diet supplemented with phytase; CS‐PRO‐PHY = canola seed diet supplemented with protease and phytase.

Abbreviation: SEM, standard error of means.

^a‐c^Means within a variable with no common superscript differ significantly (*P* < 0.05).

### 3.2 AME and Nutrient Digestibility

Table 4 shows the effect of protease and phytase enzymes in diets containing canola seed on the digestibility of AME and nutrients in broiler chickens. AME, CF and ash digestibility were higher in the control group and in broilers fed CS‐PHY and CS‐PRO‐PHY compared with birds fed the CS‐10 or CS‐PRO (*P* < 0.05). Higher CP digestibility was recorded in broilers receiving CS‐PRO and CS‐PRO‐PHY diets, compared with birds fed the control diet or the CS‐10 treatment (*P* = 0.003).

**TABLE 4:** The effect of protease and phytase enzymes in diets containing canola seed on the digestibility of AME and nutrient in broiler chickens.

Items	CON	CS‐10	CS‐PRO	CS‐PHY	CS‐PRO‐PHY	SEM	*P*‐value
AME (kcal/kg)	2863.19^a^	2686.64^b^	2664.75^b^	2853.63^a^	2862.57^a^	14.76	0.001
DM (%)	79.25	78.14	78.08	78.75	79.43	0.45	0.376
CP (%)	71.68^b^	69.23^c^	74.46^a^	70.36^b,c^	74.99^a^	0.64	0.003
EE (%)	81.73	82.75	81.83	83.71	83.74	0.70	0.114
CF (%)	33.34^a,b^	31.94^b^	32.39^b^	36.91^a^	35.92^a^	0.89	0.015
Ash (%)	58.41^a^	55.61^b^	56.63^b^	59.94^a^	59.01^a^	0.53	0.009

The experimental treatments included: CON = control diet; CS‐10 = diet containing 10% canola seed; CS‐PRO = canola seed diet supplemented with protease; CS‐PHY = canola seed diet supplemented with phytase; CS‐PRO‐PHY = canola seed diet supplemented with protease and phytase. Abbreviations: AME, apparent metabolisable energy; CF, crude fibre; CP, crude protein; DM, dry matter; EE, ether extract; SEM, standard error of means.

^a‐c^Means within a variable with no common superscript differ significantly (*P* < 0.05).

### 3.3 Digestive Enzyme Activities

The effect of protease and phytase enzymes in diets containing canola seed on digestive enzyme activities at 10 and 42 days of age is shown in Table 5. At Day 10, amylase and lipase activities were highest in broilers fed the CS‐PRO‐PHY diet, followed by the CS‐PRO, CS‐PHY and the control diet, whereas the lowest activity was observed in the CS‐10 diet (*P* = 0.001). Total protease activity at Day 10 was higher in broilers fed CS‐PRO, CS‐PHY and CS‐PRO‐PHY diets compared with those fed the CS‐10 diet (*P* = 0.027). No significant differences were detected in amylase, lipase and protease activity among treatments at Day 42 (*P* > 0.05).

**TABLE 5:** The effects of protease and phytase enzymes in diets containing canola seed on the activities of jejunum digestive enzymes of broilers.

Items	CON	CS‐10	CS‐PRO	CS‐PHY	CS‐PRO‐PHY	SEM	*P*‐value
**Amylase, IU/L**							
D10	417.99^b^	307.34^c^	445.47^b^	441.10^b^	470.87^a^	9.74	0.001
D42	516.2	513.55	525.97	522.56	517.99	17.62	0.515
**Lipase, U/L**							
D10	1332.17^b^	1150.57^c^	1299.70^b^	1321.99^b^	1452.93^a^	30.84	0.008
D42	1650.57	1669.04	1660.13	1679.65	1690.49	39.15	0.463
**Total protease, U/L**							
D10	1569.10^a^	1457.88^b^	1559.25^a^	1592.98^a^	1597.16^a^	27.67	0.027
D42	1845.96	1842.95	1850.28	1835.06	1872.14	41.55	0.695

The experimental treatments included: CON = control diet; CS‐10 = diet containing 10% canola seed; CS‐PRO = canola seed diet supplemented with protease; CS‐PHY = canola seed diet supplemented with phytase; CS‐PRO‐PHY = canola seed diet supplemented with protease and phytase.

Abbreviation: SEM, standard error of means.

^a‐c^Means within a variable with no common superscript differ significantly (*P* < 0.05).

### 3.4 Jejunum Morphology

The effect of protease and phytase enzymes in diets containing canola seed on the morphology of broiler chickens at 10 and 42 days of age is reported in Table 6. Villus height and villus‐to‐crypt ratios on Days 10 and 42 were significantly higher in broilers fed the control diet, the CS‐PHY and CS‐PRO‐PHY diets compared to the CS‐10 diet (*P* < 0.05). At Day 10, crypt depth was significantly greater in broilers fed the CS‐10 diet compared with the control treatment and CS‐PRO‐PHY diet (*P* = 0.001). At Day 42, crypt depth remained highest in the CS‐10 and CS‐PRO groups, while the lowest values were recorded in the control, CS‐PHY and CS‐PRO‐PHY treatments (*P* = 0.002).

**TABLE 6:** Effects of protease and phytase enzymes in diets containing canola seed on jejunum morphology in broilers at 10 and 42 days of age.

Items	CON	CS‐10	CS‐PRO		CS‐PHY	CS‐PRO‐PHY	SEM	*P*‐value
**Villus height, µm**								
D10	541.97^a^	510.88^b^		521.69^a,b^	544.40^a^	552.50^a^	11.38	0.001
D42	1003.02^a^	859.61^b^		910.45^a,b^	968.15^a^	1065.26^a^	25.67	0.008
**Crypt depth, µm**								
D10	58.64^b^	70.69^a^		66.60^a,b^	64.48^a,b^	58.01^b^	4.26	0.001
D42	165.25^b^	192.31^a^		195.98^a^	169.96^b^	167.23^b^	6.35	0.002
**Villus‐to‐crypt ratios**								
D10	9.24^a^	7.23^b^		7.83^b^	8.44^a,b^	9.52^a^	0.43	0.007
D42	6.07^a^	4.47^b^		4.65^b^	5.70^a^	6.37^a^	0.28	0.003

The experimental treatments included: CON = control diet; CS‐10 = diet containing 10% canola seed; CS‐PRO = canola seed diet supplemented with protease; CS‐PHY = canola seed diet supplemented with phytase; CS‐PRO‐PHY = canola seed diet supplemented with protease and phytase.

Abbreviation: SEM, standard error of means.

^a,b^Means within a variable with no common superscript differ significantly (*P* < 0.05).

### 3.5 Ileal Microbiota

Table 7 shows the effect of canola seed supplemented with phytase and protease on the ileal microbiota of broiler chickens at 10 and 42 days of age. At Day 10, *Lactobacillus* populations were highest in broilers fed the CS‐PRO‐PHY and the control diet, and lowest in the CS‐10 diet (*P* = 0.039). The counts of *C. perfringens* on Day 10 were significantly higher in broilers fed the CS‐PRO‐PHY diet compared to the CS‐10 diet (*P* = 0.014). At Day 10, *E. coli* populations were lower in the CS‐PRO‐PHY compared with the CS‐10 diet and CS‐PHY diet, which showed the highest counts (*P* = 0.006). No significant differences among treatments were detected at Day 42 (*P* > 0.05).

**TABLE 7:** Effects of protease and phytase enzymes in diets containing canola seed on ileal microbial (log_10_ CFU/g) in broilers at 10 and 42 days of age.

Items	CON	CS‐10	CS‐PRO	CS‐PHY	CS‐PRO‐PHY	SEM	*P*‐value
***Lactobacillus***							
D10	4.21^a^	3.35^b^	3.68^a,b^	3.79^a,b^	4.44^a^	0.12	0.039
D42	4.26	4.16	4.21	4.30	4.31	0.21	0.463
***C. perfringens***							
D10	3.47^a,b^	3.83^a^	3.59^a,b^	3.52^a,b^	3.19^b^	0.13	0.014
D42	4.23	4.32	4.37	4.29	4.36	0.26	0.348
***E. coli***							
D10	4.09^a,b^	4.64^a^	4.16^a,b^	4.52^a^	3.76^b^	0.18	0.006
D42	4.24	4.49	4.37	4.42	4.30	0.24	0.193

The experimental treatments included: CON = control diet; CS‐10 = diet containing 10% canola seed; CS‐PRO = canola seed diet supplemented with protease; CS‐PHY = canola seed diet supplemented with phytase; CS‐PRO‐PHY = canola seed diet supplemented with protease and phytase.

Abbreviation: SEM, standard error of means.

^a,b^Means within a variable with no common superscript differ significantly (*P* < 0.05).

### 3.6 Intestinal Digesta Viscosity

Figure 1 illustrates the effects of protease and phytase supplementation in canola‐based diets on the viscosity of intestinal contents in 10‐day‐old broilers. Viscosity in the duodenum and jejunum was significantly higher in broilers fed the CS‐10 diet compared with all other treatments (*P* < 0.01). In the ileum, viscosity was significantly lower in broilers fed the control diet, the CS‐PRO‐PHY diet and the CS‐PHY diet compared with the CS‐10 diet at Day 10 (*P* = 0.027). It should be noted that on Day 42, no significant difference in intestinal viscosity was observed between treatments, and the results were not reported numerically.

**FIGURE 1:**
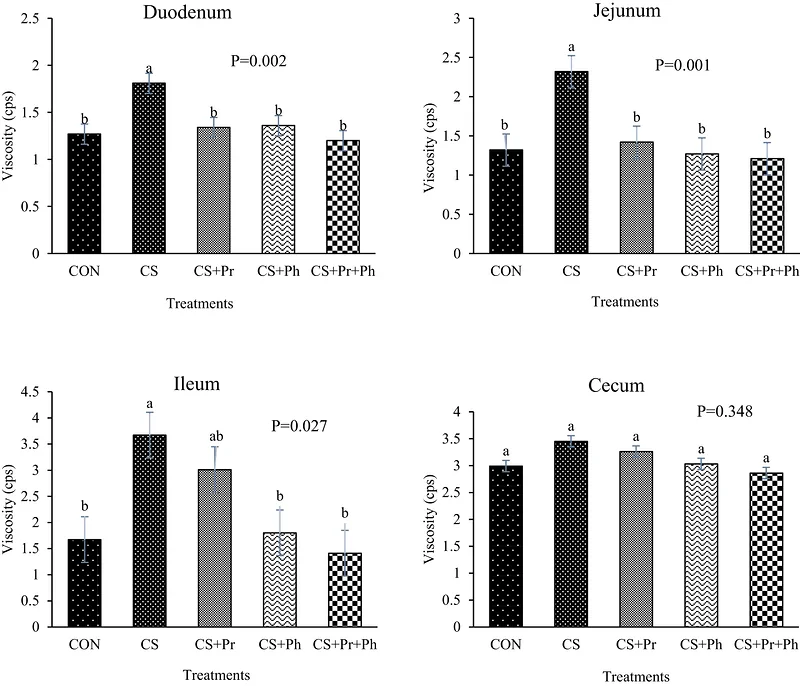
Effects of protease and phytase enzymes in diets containing canola seed on intestinal digesta viscosity in broilers at 10 days of age. The experimental treatments included: CON = control diet; CS‐10 = diet containing 10% canola seed; CS‐PRO = canola seed diet supplemented with protease; CS‐PHY = canola seed diet supplemented with phytase; CS‐PRO‐PHY = canola seed diet supplemented with protease and phytase. ^a,b^ Means within a variable with no common superscript differ significantly (*P* < 0.05).

### 3.7 Tibia Bone Mineralisation

Table 8 presents the effects of protease and phytase enzymes in diets containing canola seed on the physical and chemical characteristics of the tibia bone of broiler chickens is reported. Bone weight, bone length and the weight‐to‐length index of the bone in the CS‐PRO‐PHY treatment were significantly greater than those in the CS‐10 and CS‐PRO treatments (*P* < 0.05). The diameter of the medullary canal also showed a significant difference among treatments (*P* = 0.020), and the control treatment had a larger diameter compared with the CS‐10 treatment. Moreover, bone strength in the control, CS‐PRO‐PHY and CS‐PHY treatments was higher than in the CS‐10 treatment (*P* = 0.034). Regarding the chemical characteristics, the bone ash, calcium and phosphorus percentages were affected by the treatments, such that the use of CS‐10 compared with the control and CS‐PRO‐PHY treatments resulted in a reduction in bone ash, calcium and phosphorus (*P* < 0.05).

**TABLE 8:** Effects of protease and phytase enzymes in diets containing canola seed on tibia bone quality of broiler chickens.

Items	CON	CS‐10	CS‐PRO	CS‐PHY	CS‐PRO‐PHY	SEM	*P*‐value
**Physical characteristics**							
Weight (g)	6.92^a,b^	5.92^b^	5.93^b^	7.00^a,b^	7.58^a^	0.23	0.012
Length (mm)	99.70^a,b^	93.74^c^	97.02^b,c^	99.43^a,b^	102.26^a^	0.74	0.004
Diaphysis diameter (mm)	8.54	7.84	7.64	8.38	8.39	0.16	0.077
Medullary canal diameter (mm)	4.61^a^	3.48^b^	3.62^a,b^	4.11^a,b^	4.29^a,b^	0.20	0.020
Mid‐wall thickness (mm)	1.96	2.11	2.08	2.13	2.05	0.09	0.943
Weight/length index	69.15^a,b^	63.10^b^	61.07^b^	70.43^a,b^	74.13^a^	2.04	0.048
Strength (N)	4278.7^a^	3074.0^b^	3535.0^a,b^	4296.0^a^	4055.7^a^	72.29	0.034
Robusticity index	5.28	5.23	5.40	5.23	5.25	0.32	0.143
Tibiotarsal index	46.38	53.59	54.24	50.86	49.03	3.28	0.531
**Chemical characteristics**							
Ash (%)	41.76^a^	39.50^b^	40.03^a,b^	40.93^a,b^	41.93^a^	0.43	0.017
Calcium (%)	15.54^a^	14.39^b^	14.68^a,b^	14.96^a,b^	15.57^a^	0.25	0.006
Phosphorus (%)	5.55^a^	5.11^b^	5.32^a,b^	5.53^a^	5.57^a^	0.11	0.008

The experimental treatments included: CON = control diet; CS‐10 = diet containing 10% canola seed; CS‐PRO = canola seed diet supplemented with protease; CS‐PHY = canola seed diet supplemented with phytase; CS‐PRO‐PHY = canola seed diet supplemented with protease and phytase.

Abbreviation: SEM, standard error of means.

^a‐c^Means within a variable with no common superscript differ significantly (*P* < 0.05).

## 4 Discussion

### 4.1 Performance

The results of the present study demonstrated that partial replacement of soybean meal with 10% canola seed, without exogenous enzyme supplementation, led to reduced FI, decreased BWG and ultimately impaired FCR in broiler chickens. These findings are consistent with previous reports indicating that inclusion of canola products, particularly in the form of whole seeds, is associated with reduced growth performance due to the presence of anti‐nutritional factors and limitations in digestibility (Keyhani et al. 2024; Souza et al. 2025; Kandel et al. 2025b). Canola seeds contain substantial amounts of phytate, CF, lignin and glucosinolates, which can restrict FI and growth performance by decreasing diet palatability, increasing intestinal digesta viscosity and reducing the bioavailability of nutrients (Toghyani et al. 2017). The observed reduction in FI in the canola‐only treatment is considered a primary factor contributing to the lower BWG. As noted by Kandel et al. (2025b), when nutrient digestibility and FI remain unchanged, decreased FI is likely the main driver of reduced growth performance in diets containing high levels of canola products. In the present study, the decrease in FI in the canola treatment was likely due to the combined effects of high phytate and fibre levels on appetite and feeding behaviour.

Supplementation of canola‐based diets with protease improved BWG and FI, although its effect on FI was less pronounced compared with phytase. This outcome can be attributed to the primary mode of action of protease, which mainly enhances dietary protein hydrolysis and improves amino acid absorption (Vieira et al. 2023). Increased availability of absorbable amino acids promotes body protein synthesis, thereby enhancing weight gain without necessarily increasing FI (Dosković et al. 2013). These findings align with previous studies by Zamir et al. (2021) and Cowieson et al. (2017), who reported that protease primarily improves protein utilisation efficiency rather than FI. In contrast, phytase supplementation had a more pronounced effect on FI and consequently BWG. By hydrolysing phytate, phytase releases phosphorus and enhances the bioavailability of calcium, protein, amino acids and energy (Kies et al. 2001). Reducing the anti‐nutritional effects of phytate and protein–phytate complexes likely improved diet palatability and increased feed consumption (Woyengo and Nyachoti 2013). Enhanced FI, combined with improved nutrient utilisation, ultimately contributed to the increased BWG and improved FCR observed in the phytase‐supplemented groups. These results are consistent with previous reports by Derakhshan et al. (2023) and Moradi et al. (2023) demonstrating the positive effects of phytase on growth performance. The greatest improvement in growth performance parameters, including BWG and FI, was observed in broilers fed the diet supplemented with both protease and phytase. This superior response can be attributed to the synergistic interaction between the two enzymes. Phytase reduces structural and chemical barriers to protein accessibility by hydrolysing phytate, thereby creating more favourable conditions for protease activity (Borda‐Molina et al. 2019). Protease, in turn, maximises the utilisation of nutrients released by phytase by enhancing the rate and efficiency of protein digestion (He et al. 2026). This positive interaction between phytase and protease has been previously reported by Borda‐Molina et al. (2019), who demonstrated that phytase can increase the effectiveness of protease in improving amino acid digestibility. In the present study, the observed improvements in growth performance are likely associated with the combined effects of enhanced nutrient utilisation and improved intestinal function, including villus morphology, microbial populations, digestive enzyme activity and reduced digesta viscosity. Increased digestive enzyme activity probably enhanced the breakdown of dietary substrates (Yi et al. 2024), while reduced intestinal viscosity improved nutrient flow and enzyme–substrate interactions (Matthiesen et al. 2021). Collectively, these physiological and microbial adaptations likely created a more efficient digestive environment, ultimately resulting in improved feed efficiency, higher BWG and superior overall growth performance.

### 4.2 AME and Nutrient Digestibility

The improvements in AME and nutrient digestibility observed in broilers fed canola‐based diets supplemented with protease and/or phytase can be explained by multiple interrelated mechanisms. Phytase hydrolyses phytate, releasing phosphorus and other bound minerals, which not only enhances energy availability but also reduces the anti‐nutritional effects of phytate on protein and carbohydrate digestion (Zeller et al. 2015). Protease supplementation, on the other hand, enhances the hydrolysis of dietary proteins, thereby increasing the digestibility of CP and amino acids (Mahmood et al. 2017). The combination of both enzymes likely produced additive or synergistic effects, as reflected in the increased AME, CF and ash digestibility. Improved nutrient digestibility is also closely linked to intestinal morphology and microbial balance. In the present study, broilers receiving enzyme‐supplemented diets exhibited higher villus height and villus‐to‐crypt ratios, which likely increased absorptive surface area and nutrient uptake efficiency. Additionally, the reduction in ileal and jejunal viscosity, along with favourable shifts in microbial populations, likely facilitated more efficient enzymatic action and nutrient absorption, contributing to the observed improvements in AME and nutrient digestibility. These findings align with previous studies reporting that exogenous enzymes can mitigate the negative effects of anti‐nutritional factors in canola and other high‐fibre ingredients (Mushtaq et al. 2007; Rezaeipour et al. 2015; Toghyani et al. 2017; Elbaz et al. 2023; Kandel et al. 2025b). Toghyani et al. (2017) and Kandel et al. (2025b) observed that phytase and protease supplementation increased protein and energy digestibility in broilers fed canola or canola meal, supporting the results of the current study. Moreover, the observed correlations between reduced intestinal viscosity, enhanced villus morphology and improved nutrient digestibility are consistent with findings by Papadopoulos et al. (2018) and Hafeez et al. (2026), who demonstrated that lower digesta viscosity and a balanced gut microbiota enhance enzymatic efficiency and feed utilisation.

### 4.3 Digestive Enzyme Activities

The current study indicated that supplementation of canola seed‐based diets with protease and phytase, either individually or in combination, enhanced digestive enzyme activity in the jejunum of young broilers, whereas by 42 days of age, differences among treatments were no longer evident. These observations are consistent with previous findings suggesting that exogenous enzymes have the most pronounced effects on digestive function during the early growth phase when the endogenous enzyme system is still immature (Cowieson and Kluenter 2019; Vieira et al. 2025). Similar studies have reported that protease and phytase supplementation can increase amylase, lipase and protease activities in broilers fed diets with high levels of anti‐nutritional factors, such as phytate or non‐starch polysaccharides, leading to improved nutrient hydrolysis and utilisation (Jiang et al. 2020; Radhi et al. 2023; Zakria et al. 2025). The diminished response in older birds (42 days) is likely due to maturation of their digestive system, with endogenous enzyme secretion reaching a functional plateau that diminishes the relative impact of exogenous supplementation (Vertiprakhov et al. 2022). The observed enhancement of digestive enzyme activity in the early growth phase can be mechanistically explained by multiple, interrelated factors. Exogenous protease likely facilitated hydrolysis of dietary proteins and protein–phytate complexes, increasing the availability of absorbable amino acids and peptides, which in turn may stimulate endogenous enzyme secretion through feedback mechanisms (Almeida et al. 2025). Phytase, by breaking down phytate, not only increased the release of phosphorus and bound minerals but also reduced the inhibitory effects of protein–phytate complexes on enzymatic digestion (Bernardes et al. 2022). Additionally, both enzymes may have contributed to reducing digesta viscosity and mitigating the encapsulating effects of fibre and other anti‐nutritional factors in canola seeds, thereby enhancing substrate accessibility for endogenous enzymes (Kandel et al. 2025b). These mechanisms explain how early enhancement of digestive enzyme activity supports improved nutrient digestibility and feed efficiency, contributing to the superior growth performance observed in enzyme‐supplemented broilers.

### 4.4 Jejunum Morphology

The present study demonstrated that supplementation of canola seed diets with protease and phytase, either alone or in combination, positively influenced jejunal morphology in broilers. Broilers fed enzyme‐supplemented diets exhibited higher villus height and villus‐to‐crypt ratios compared to birds receiving the canola‐only diet, indicating improved absorptive capacity and intestinal health. These findings align with previous research showing that exogenous enzymes can mitigate the negative effects of anti‐nutritional factors, such as phytate and fibre, on intestinal structure (Disetlhe et al. 2017; Akram et al. 2025). By reducing intestinal viscosity and partially breaking down indigestible compounds, protease and phytase appear to promote a more favourable environment for enterocyte proliferation, leading to taller villi and optimised villus‐to‐crypt ratios (Olfati et al. 2021). Conversely, diets containing canola without enzyme supplementation were associated with increased crypt depth, likely reflecting higher epithelial turnover in response to suboptimal nutrient availability and intestinal stress (Hollemans et al. 2020). Mechanistically, the enhancement in jejunal morphology can be attributed to the combined effects of improved nutrient digestibility, reduced anti‐nutritional constraints and more efficient enzymatic hydrolysis of proteins and complex carbohydrates. Protease increases the liberation of amino acids and peptides, which serve as substrates for enterocyte growth (Cardinal et al. 2019), while phytase reduces the inhibitory effects of phytate on nutrient absorption and mineral availability (Zeller et al. 2015). These structural improvements may also interact synergistically with the intestinal microbiota, as favourable populations of *Lactobacillus* and reduced counts of *E. coli* and *C. perfringens* can support epithelial integrity and villus development (Kasayizadeh et al. 2025).

### 4.5 Ileal Microbiota

Broilers fed the combination of protease and phytase exhibited higher *Lactobacillus* counts and lower *E. coli* populations compared to those receiving the canola‐only diet, suggesting an improved intestinal microbial balance. These findings are consistent with previous reports demonstrating that exogenous enzymes can reduce the adverse effects of canola products on gut microbiota by enhancing nutrient availability and reducing substrate for pathogenic bacteria (De Vries et al. 2014; Elbaz et al. 2023). In contrast, the canola‐only diet, containing higher levels of indigestible fibre and phytate, provided a less favourable environment for beneficial microbes (Inglis et al. 2021), leading to reduced *Lactobacillus* populations and higher counts of potentially pathogenic bacteria such as *E. coli*. Mechanistically, the observed microbial shifts may be attributed to the enhanced digestibility and absorption of nutrients, which limit the availability of undigested substrates that favour the growth of opportunistic pathogens (Yang et al. 2008). Protease supplementation increases the release of amino acids and peptides that can be utilised by beneficial bacteria (Huyan et al. 2022), while phytase reduces phytate content, improving mineral availability and reducing gut viscosity (Anwar et al. 2023). Lower intestinal viscosity promotes better nutrient flow and microbial habitat conditions, further supporting the proliferation of *Lactobacillus* and the suppression of *E. coli* and *C. perfringens* (Latorre et al. 2015).

### 4.6 Intestinal Digesta Viscosity

Intestinal digesta viscosity is a critical determinant of nutrient absorption and gastrointestinal efficiency in broilers, particularly during the early growth phase (Yasar 2003). High viscosity, often induced by soluble fibres and anti‐nutritional factors such as phytate in canola seeds, can impede the flow of digesta and reduce the accessibility of nutrients to digestive enzymes (Bell 1993). The observed reductions in viscosity with enzyme supplementation likely reflect the ability of protease and phytase to degrade protein–phytate complexes and soluble fibre components, facilitating more efficient mixing of digesta and enhancing nutrient diffusion along the small intestine (Selle et al. 2023). This mechanistic link between reduced viscosity and improved nutrient accessibility aligns with previous reports indicating that high digesta viscosity restricts enzymatic hydrolysis and absorption, ultimately limiting feed efficiency and growth in young broilers (Amerah et al. 2008; Murphy et al. 2009). Phytase primarily acts by hydrolysing phytate, which not only releases bound phosphorus and minerals but also disrupts viscous complexes that otherwise hinder enzymatic action and nutrient uptake (Abbasi et al. 2019). Protease contributes by breaking down dietary proteins and peptide–fibre complexes, further reducing luminal viscosity and improving the exposure of substrates to endogenous enzymes (Olfati et al. 2021). The complementary effects of these enzymes create a more favourable digestive environment, where the mobility of digesta, interaction between enzymes and substrates and absorption of energy and amino acids are enhanced (Kandel et al. 2025b). Studies by Yuan et al. (2008) and Attia et al. (2022) support this synergistic concept, demonstrating that enzyme combinations can optimise the availability of nutrients by altering gut physicochemical properties rather than simply increasing FI. These interactions highlight that modulating gut viscosity through targeted enzyme supplementation is a key strategy for enhancing the functional capacity of the digestive system and maximising feed utilisation in broilers.

### 4.7 Tibia Bone Mineralisation

The results of the present study indicated that the dietary inclusion of 10% canola seed without enzyme supplementation impaired the physical (weight, length and strength) and chemical (ash, calcium and phosphorus percentages) characteristics of the tibia in broiler chickens. These findings are in agreement with those of Hafeez et al. (2025), who reported that canola‐based diets could significantly reduce tibia weight and robusticity indices in broilers. In our study, this impairment was clearly reflected in the decreased bone breaking strength and ash content in the non‐enzyme canola group, indicating compromised bone mineralisation due to the presence of anti‐nutritional factors in canola seeds (Khajali and Slominski 2012). Conversely, dietary supplementation with the protease and phytase combination (CS‐PRO‐PHY) effectively restored these parameters to levels comparable to the control group. This highlights a critical distinction between simple single‐enzyme approaches and comprehensive enzyme combinations. In this regard, Disetlhe et al. (2017) observed that single‐enzyme supplementation alone might not suffice to prevent bone abnormalities, such as rickets, in birds fed canola‐based diets, suggesting that managing the complex anti‐nutritional profile of canola requires more advanced strategies. The findings of the current study suggest that the combined application of protease and phytase successfully overcame the nutritional constraints of canola seed, performing superiorly to the single‐enzyme treatments. From a physiological perspective, the improvements in bone mineral chemistry (ash, calcium and phosphorus percentages) under enzyme‐supplemented treatments can be primarily attributed to the hydrolysis of phytate by phytase, which liberates phytate‐bound phosphorus (Beeson et al. 2017). Phytate in canola seed forms insoluble complexes with essential minerals, severely limiting their bioavailability (Parsons and Rochell 2024). In the present study, the enhanced ash digestibility and AME observed in phytase‐containing groups provide indirect evidence of increased mineral availability for bone mineralisation. Furthermore, the development of the organic bone matrix (principally collagen and structural proteins) plays a vital role in skeletal integrity (Šromová et al. 2023). Bone consists of a structural framework of organic matrix; thus, the improved crude protein digestibility in protease‐supplemented groups observed in our trial likely enhanced amino acid availability for collagen matrix synthesis, providing a more stable template for calcium and phosphorus deposition. The significant reduction in the medullary canal diameter of the non‐enzyme canola group compared with the control further confirms that the unsupplemented canola diet failed to support optimal bone structural development.

## 5 Conclusion

The present study clearly demonstrates that partial replacement of soybean meal with 10% canola seed in broiler diets can negatively impact performance and tibia bone mineralisation, primarily due to anti‐nutritional factors and limited nutrient accessibility. However, supplementation with exogenous enzymes, particularly the combination of protease and phytase, effectively mitigates these limitations by reducing digesta viscosity, enhancing digestive enzyme activity, improving villus‐to‐crypt ratios, promoting a more favourable ileal microbial profile and improving nutrient digestibility. Importantly, the study underscores that while protease primarily enhances protein utilisation, phytase exerts a more pronounced effect on viscosity, villus height and overall nutrient availability and their combination delivers superior performance outcomes.

## Author Contributions

**Ramazan Sheikh‐Nazari**: investigation, writing – original draft preparation, methodology, visualisation. **Kaveh Jafari‐Khorshidi**: project administration, validation, reviewing, visualisation, supervision, funding acquisition. **Mohammad Ali Jafari**: resources, data analysis, writing – review and editing.

## Funding

The authors have nothing to report.

## Conflicts of Interest

The authors declare no conflicts of interest.

## Ethics Statement

All experimental procedures involving animals were reviewed and approved by the Animal Care and Animal Research Ethics Committee of the Islamic Azad University, Sari Branch, Iran (Approval No. IR.IAU.SARI.REC.1404.038), and were conducted in accordance with the European Union regulations governing the care and use of animals for scientific purposes (European Parliament, 2010).

## Data Availability

The data that support the findings of this study are available from the corresponding author, Kaveh Jafari‐Khorshidi, upon reasonable request.
